# Targeting the AKT/mTOR axis: pectolinarigenin induces autophagy and apoptosis in human cervical cancer cells

**DOI:** 10.3389/fphar.2026.1544170

**Published:** 2026-03-17

**Authors:** Yaoyao Fang, Jing Bai, Sijia Bo, Xiaoli Cui, Haiping Song, Li Guo, Zhaohui Luan, Qixuan Sui, Yingchun Zheng, Li Sun

**Affiliations:** 1 The First Affiliated Hospital of Hainan Medical University, Hainan Medical University, Haikou, China; 2 School of Clinical Medicine, Shandong Second Medical University, Weifang, China; 3 Department of Maternity, Jinan Maternity and Child Care Hospital Affiliated to Shandong First Medical University, Jinan Maternity and Child Care Hospital, Jinan, China; 4 First Department of Gynecology Oncology, Qingdao Central Hospital, University of Health and Rehabilitation Sciences (Qingdao Central Hospital), Qingdao, China; 5 Department of Gynecology, Pingdu People’s Hospital, Shandong, Pingdu, China; 6 Qingdao Hospital, University of Health and Rehabilitation Sciences (Qingdao Municipal Hospital), Qingdao, China

**Keywords:** Akt/mTOR signaling pathway, apoptosis and autophagy, cervical cancer, network pharmacology, pectolinarigenin

## Abstract

Cervical cancer (CC) remains a significant global health issue, accounting for approximately 7% of all cancer cases in women. This study investigated the anti-cancer potential of pectolinarigenin (PEC), a bioactive compound derived from plants, aiming to explore its therapeutic effects and underlying mechanisms against CC. By integrating network pharmacology analysis with cellular assays, we identified 13 key targets of PEC related to CC, with molecular docking highlighting AKT as a primary target. Experimentally, PEC demonstrated strong anti-cancer effects on cervical cancer both *in vivo* and *in vitro*. Western blotting analysis revealed that PEC treatment led to a dose-dependent decrease in Bcl-2 protein levels, coupled with increased activation of pro-apoptotic markers Bax and cleaved caspase-3 in both cell lines. PEC also elevated the levels of LC3B II protein, indicating the induction of autophagy. Notably, this autophagic response was inhibited by 3-MA, an autophagy inhibitor, suggesting that PEC played a regulatory role in activating autophagy. Mechanistic studies confirmed that PEC effectively suppressed the AKT/mTOR signaling pathway, a critical regulator of both autophagy and apoptosis in cancer cells. Overall, this is the first study to demonstrate that PEC exerted potent anti-cancer effects against CC by concurrently inducing autophagy and apoptosis through targeted inhibition of the AKT/mTOR pathway. These findings highlighted the potential of PEC as a promising natural therapeutic agent for CC, paving the way for new treatment strategies. Further comprehensive research is warranted to fully explore PEC’s therapeutic capabilities and to develop innovative anti-cancer therapies.

## 1 Introduction

Cervical cancer (CC) ranks as the fourth most prevalent cancer among women globally and is the second leading cause of cancer-related mortality in women aged 20–39 years (Siegel et al., 2023). The primary etiological factor for CC is chronic infection with high-risk human papillomavirus (HPV) (Ferrall et al., 2021), with HPV types 16 and 18 accounting for over 70% of all CC cases (Ferrall et al., 2021; de Martel et al., 2017). While surgical resection and radiotherapy remain the standard treatments for early-stage CC, the combination of chemotherapy with radiotherapy has significantly improved survival outcomes (Gopu et al., 2021). Despite these advancements, the variability in treatment responses often leads to poor prognosis and reduced survival rates in many patients. Hence, there is an urgent need to identify novel molecular targets and develop innovative therapeutic strategies to enhance the management of CC.

Autophagy and apoptosis are two fundamental programmed cell death pathways that play critical roles in cancer biology (Das et al., 2021). A wealth of studies has demonstrated that activating these pathways can effectively hinder the progression of CC (Liu et al., 2024a; Liu et al., 2022). Apoptosis, a caspase-dependent programmed cell death mechanism, is activated by genotoxic stressors, therapeutic agents, or survival signal withdrawal (Xu et al., 2019; Carneiro and El-Deiry, 2020). Execution proceeds via mitochondrial (Bcl-2 family-mediated MOMP) or extrinsic (death receptor oligomerization) pathways (Carneiro and El-Deiry, 2020; Obeng, 2021; Morana et al., 2022). Physiologically, apoptosis serves as a crucial defense mechanism, facilitating the elimination of malignant cells and thus inhibiting tumor development (Nguyen et al., 2023).

Autophagy, another essential cellular process, is responsible for the degradation of long-lived proteins, damaged organelles, and misfolded proteins within eukaryotic cells (Levy et al., 2017; Deng et al., 2023). Impaired autophagy has been implicated as a significant oncogenic factor and is recognized as a potential therapeutic target in several human cancers, including CC (Mattoscio et al., 2018). The regulation of autophagy involves intricate molecular pathways, notably including sequestosome-1 (SQSTM1/p62), microtubule-associated light chain 3 (LC3), and the mammalian target of rapamycin (mTOR) signaling axis (Liu et al., 2022). Novel therapeutic strategies may be focused at targeting autophagy and apoptosis, which may be achieved by small molecule modulators or gene editing technologies, etc (Liu et al., 2024b; Emdad et al., 2020). These approaches can effectively reprogram cancer cells, inducing cell death by either triggering apoptotic pathways or activating cytotoxic autophagy (Emdad et al., 2020).

Pectolinarigenin (PEC), a bioactive flavone isolated from medicinal plants including Citrus species and thistle, demonstrates multifaceted therapeutic potential (Cheriet et al., 2020). Its pharmacological profile encompasses anti-inflammatory, immunomodulatory, and antioxidant activities (Cheriet et al., 2020; Heimfarth et al., 2021; Qian et al., 2024; Li et al., 2023a). Mechanistic studies reveal PEC selectively targets topoisomerase IIα (TOP2A), inducing DNA damage and G2/M phase arrest in bladder cancer models (Deng et al., 2023). Additionally, antiproliferative efficacy has been further documented in osteosarcoma, melanoma, and breast carcinoma (Gan et al., 2019; Zhang et al., 2022; Deng et al., 2020). However, its therapeutic potential and the precise mechanisms of action in CC remain largely unexplored.

With the rapid expansion of biological databases, network pharmacology has emerged as a cutting-edge approach in drug discovery (Hopkins, 2008). This methodology shifts the research paradigm from the traditional “single drug-single target” model to a holistic framework that integrates multiple targets within complex drug-disease gene signaling pathways (Huang et al., 2014). In this study, we utilized a network pharmacology approach to identify potential therapeutic targets and unravel the mechanisms of PEC in CC. To further substantiate these findings, we conducted both *in vitro* and *in vivo* experiments to validate the effects of PEC on CC cells. Through this comprehensive analysis, we aimed to shed light on the anti-cancer properties of PEC and introduce it as a novel candidate for targeted therapy against CC.

## 2 Materials and methods

### 2.1 Materials and reagents

PEC, with a chemical formula of C_17_H_14_O_6_ and a PubChem CID of 5320438, was procured from MedChemExpress (MCE, China), boasting a purity of over 99.59% (Catalog no. HY-N0493). PEC was dissolved in dimethyl sulfoxide (DMSO), sourced from Solarbio (Beijing, China). Phosphate-buffered saline (PBS; MA0015) was purchased from Meilunbio (DaLian, China). The autophagy inhibitor 3-Methyladenine (3-MA), crystal violet, and the phosphatidylinositol-3-kinase (PI3K) inhibitor LY294002 were also obtained from Solarbio Life Sciences. For cell culture, Dulbecco’s Modified Eagle Medium (DMEM; MA0212) and penicillin/streptomycin (P/S; MA0110) were purchased from Meilunbio (DaLian, China). Fetal bovine serum (FBS; FSP-500) was acquired from Excell Bio (Shanghai, China). The CCK-8 Assay Kit was supplied by MCE (Shanghai, China), while the EdU Apollo-567 Kit and TUNEL Apoptosis Kit (Green) were sourced from RiboBio (Guangzhou, China). The FITC Annexin V Apoptosis Detection Kit was obtained from BD Pharmingen (United States).

For protein quantification, the BCA Protein Quantitation Kit was procured from Solarbio (Beijing, China). Primary antibodies used in Western blotting analysis included β-actin (ab8226; 1:1,000), total AKT (ab8805; 1:1,000), and GAPDH (181602; 1:1,000) from Abcam (United Kingdom). Additional antibodies for LC3A/B (14600-1-AP; 1:5,000), p62 (18420-1-AP; 1:5,000), Bcl-2 (12789-1-AP; 1:2,000), Bax (50599-2-Ig; 1:5,000), phospho-mTOR (67778-1-Ig; 1:5,000), and mTOR (66888-1-Ig; 1:5,000) were sourced from Proteintech (Wuhan, China). Phospho-AKT (Thr308; 13038S; 1:1,000) and cleaved-caspase 3 (6,322; 1:1,000) were purchased from Cell Signaling Technology (CST, United States). Secondary antibodies, including anti-rabbit IgG (SA00001-2; 1:3,000), were supplied by Proteintech (Wuhan, China).

### 2.2 Network pharmacology

#### 2.2.1 Identification of potential target proteins for PEC and CC

To identify the potential target proteins of PEC in humans, several online databases were utilized, including SwissTargetPrediction (http://swisstargetprediction.ch/). Disease-related targets specific to CC were retrieved from various databases such as GeneCards (https://www.genecards.org/) and OMIM (https://omim.org/). After aggregating the target lists, redundant entries were removed to obtain a refined set of CC-related targets. Further validation and refinement of these targets were performed using the UniProt database (https://www.uniprot.org/), with the focus restricted to human-specific (*Homo sapiens*) genes.

#### 2.2.2 Construction of protein-protein interaction (PPI) networks and core target identification

To determine the overlapping targets between PEC and CC, a comparative analysis was conducted using the Venny 2.1 tool (https://bioinfogp.cnb.csic.es/tools/venny).

The identified potential targets of PEC for CC were uploaded to the STRING database (https://cn.string-db.org), with the organism specified as “*H. sapiens*”. An interaction score threshold of 0.4 was applied to filter significant interactions. Subsequent PPI analysis was conducted using default settings. PPI networks for PEC and CC targets were generated using the BisoGenet plugin in Cytoscape, with target data retrieved from publicly available databases. Merged PEC and CC networks revealed a shared subnetwork of overlapping molecular interactions. Topological analysis (degree, betweenness/closeness centrality) via CytoNCA identified hub nodes (degree ≥2× median network value). A three-step screening protocol isolated critical regulatory nodes meeting or exceeding median thresholds across all three centrality parameters. Through the aforementioned steps, we have successfully identified the key targets of PEC in cervical cancer.

#### 2.2.3 Gene Ontology (GO) and Kyoto encyclopedia of genes and genomes (KEGG) pathway enrichment analysis

GO and KEGG pathway enrichment analyses were performed using the DAVID 6.7 database (https://david.ncifcrf.gov/). For the KEGG pathway analysis, pathways with a p-value ≤0.05 and a Benjamini value ≤0.05 were considered statistically significant, with only the top 15 pathways (ranked by count values) included in the final analysis. The GO enrichment analysis encompassed three key categories: cellular component, molecular function, and biological process. To effectively illustrate the enriched pathways and biological processes, the results were visualized using a bioinformatics platform (https://www.bioinformatics.com.cn/) to generate bubble plots and histograms. Finally, a target-pathway network mapping PEC’s therapeutic mechanisms against CC was reconstructed in Cytoscape software (version 3.9.2).

#### 2.2.4 Molecular docking validation

To predict the binding affinities between PEC and core target proteins implicated in CC, molecular docking simulations were carried out using AutoDockTools 1.5.6 and AutoDock Vina, following the methodology outlined by Eberhardt et al., (2021). The docking procedure involved several key stages:

Ligand Preparation: The structural file for PEC was initially retrieved in sdf format from the PubChem database as described by Kim et al. (Kim et al., 2021) and converted to mol2 format. Using ChemBio3D Ultra 14.0, the structure underwent 3D conversion, followed by energy minimization using the MM2 force field. The optimized structure was saved in pdb format and subsequently prepared in AutoDockTools 1.5.6, where hydrogen atoms were added, and the file was saved as a pdbqt format for further docking analysis.

Protein Preparation: Crystal structures of the core target proteins were sourced from the Protein Data Bank (PDB) using gene names as search identifiers. PyMOL 2.4.1 software was employed to remove water molecules and any co-crystallized ligands. The refined protein structures were then prepared in AutoDockTools 1.5.6 by adding hydrogens, calculating charges, and assigning atom types, after which they were saved in pdbqt format.

Grid Box Setup: The identification of potential binding sites was performed using DoGSiteScorer (https://proteins.plus/). The grid box dimensions and coordinates were adjusted accordingly to encompass these predicted binding sites, ensuring an optimal fit for the molecular docking analysis.

Molecular Docking and Interaction Analysis: Docking simulations were executed using AutoDock Vina to evaluate the binding energies between PEC and the target proteins. Visualization and interaction analysis of the docked complexes were conducted using PyMOL 2.4.1 and Discovery Studio 2024, providing detailed insights into the binding interactions between PEC and key target proteins.

### 2.3 Experimental section

#### 2.3.1 Cell culture

CC cell lines (HeLa and SiHa) were cultured in complete DMEM, which was supplemented with 10% FBS and 1% P/S solution. The cells were maintained at 37 °C in a humidified atmosphere containing 5% CO_2_. Upon reaching 80%–90% confluence, the cells were subcultured or cryopreserved for future experiments.

#### 2.3.2 Cell viability and proliferation assay

HeLa and SiHa cells were seeded at a density of 6 × 10^3^ cells per well in 96-well plates and allowed to adhere for 12 h. Cells were treated with graded concentrations of PEC and analyzed at 24, 48, and 72 h post-treatment. Following medium replacement with serum-free DMEM containing 10% CCK-8 reagent (MCE, HY-K0301), cells were incubated for 2 h at 37 °C. Absorbance was measured at 450 nm using a microplate reader with triplicate measurements. For proliferation, cells treated with PEC for 24 h were incubated with EdU working solution (1:1,000, Apollo-567 Kit, Ribobio) for 2 h, fixed with 4% paraformaldehyde, permeabilized with Triton X-100, stained with Apollo solution (30 min, dark), and counterstained with DAPI (C0065, Solarbio). EdU-positive cells were quantified via fluorescence microscopy.

#### 2.3.3 Colony formation assay

HeLa and SiHa cells were harvested and plated into 6-well plates at a density of 500 cells per well. The cells were treated with different concentrations of PEC (0, 5, 10, 25, and 50 μM) for seven consecutive days, with the culture medium replaced every 3 days. Following colony formation, culture medium was aspirated followed by gentle rinsing with 1 mL PBS per well. After complete PBS removal, cell fixation was performed using 4% paraformaldehyde (2 mL/well; Anhui, China; BL539A) at room temperature for 30 min. Following fixation, cells underwent two PBS washing cycles to remove residual fixative. For colony visualization, fixed colonies were stained with 1 mL of 1% crystal violet solution (Solarbio, Shanghai, China; Cat# 1062) per well under ambient conditions for 15 min. Excess stain was removed through three consecutive PBS washes (1 mL/well, 2 min per wash). Finally, air-dried specimens were photographed for observation. All experimental steps were performed in triplicate unless otherwise specified.

#### 2.3.4 TUNEL assay

Apoptosis in HeLa and SiHa cells was analyzed using the TUNEL Apoptosis Kit (Green). Briefly, cells (6 × 10^3^/well in 96-well plates) were treated for 24 h, fixed with 4% paraformaldehyde (30 min), permeabilized with 0.1% Triton X-100/0.1% sodium citrate (10 min, RT), and incubated with TUNEL reaction mixture (37 °C, 2 h, dark). Nuclei were counterstained with DAPI. Apoptotic cells were quantified and visualized using fluorescence microscopy.

#### 2.3.5 Cell apoptosis assay

HeLa and SiHa cells were seeded at a density of 5 × 10^5^ cells per well in 6-well plates and incubated for 12 h. Following this, the culture medium was treated with different concentrations of PEC (0, 5, 10, 25, and 50 μM) for 24 h. The cells were then harvested, washed twice with PBS, and resuspended in 500 μL of binding buffer. The cells were stained with Annexin V-FITC in the dark at room temperature for 15 min. Prior to flow cytometry analysis, propidium iodide (PI) solution was added. Apoptosis was evaluated using the Annexin V-FITC/PI detection kit (BD Pharmingen, United States), followed by analysis with flow cytometry.

#### 2.3.6 Wound healing assay

For the wound healing assay, HeLa and SiHa cells were plated at a density of 5 × 10^5^ cells per well in 6-well plates and incubated for 12 h to achieve a confluent monolayer. A scratch was gently created on the cell surface using a sterile pipette tip. Fresh medium containing various concentrations of PEC was added, and images were captured at 0 h, 24 h, and 48 h post-treatment to assess the extent of cell migration and wound closure.

#### 2.3.7 Transwell assay

For the Transwell invasion assay, a total of 2,000 cells were counted and resuspended in 200 μL of serum-free medium mixed with Matrigel, and then seeded into the upper chamber of the Transwell insert. The lower chamber was filled with 600 μL of complete medium as a chemoattractant. After 24 h of incubation, the cells that had invaded through the membrane were fixed with 500 μL of paraformaldehyde and stained with crystal violet. The invasive cells on the lower side of the membrane were visualized and imaged using an optical microscope. The relative invasion rates were quantified and compared against the control group.

#### 2.3.8 JC-1 staining detection

HeLa and SiHa cells (logarithmic phase) were seeded in 6-well plates (3 × 10^4^ cells/mL, 2 mL/well) and cultured overnight. Cells were treated with polyelectrolyte complexes (PECs) at different concentrations for 24 h. Post treatment, 500 μL cell suspension was stained with 10 µL 5X JC-1 buffer (37 °C, 20 min), washed twice, then analyzed using an AGreen ELISA reader: 485 nm (green/monomer) and 535 nm (red/aggregate). Membrane staining patterns were qualitatively assessed across concentrations.

#### 2.3.9 Western blotting analysis

Total protein lysates were extracted from HeLa and SiHa cells using RIPA lysis buffer (Solarbio, China) supplemented with PMSF and phosphatase inhibitors to prevent protein degradation. The protein concentrations were measured with the BCA Protein Quantitation Kit. Equal amounts of protein samples were denatured by boiling at 100 °C for 10 min in a loading buffer (Solarbio, China). Proteins were separated on SDS-PAGE gels (7.5%, 10% and12%) and transferred onto polyvinylidene difluoride (PVDF) membranes (Meilunbio, Dalian, China). The membranes were blocked and then incubated overnight at 4 °C with specific primary antibodies, including those for total-AKT, phospho-AKT (Thr473), Bcl-2, Bax, cleaved caspase-3, mTOR, phospho-mTOR, β-actin, and GAPDH. The following day, the membranes were incubated with horseradish peroxidase (HRP)-conjugated secondary antibodies at room temperature for 1 h. The immunoreactive bands were detected using the Immobilon Western Chemiluminescent HRP Substrate (Beyotime, China).

#### 2.3.10 Mice xenograft models

All procedures involving animal care and experimentation were carried out in accordance with the guidelines and with the approval of the Animal Research Ethics Committee at Qingdao Technology University (Permit No. QKDLL-2025-150). Four-week-old female BALB/c nude mice (15 ± 3 g), bred in the school laboratory Animal Center, were maintained in a specific pathogen-free facility under controlled conditions of temperature (22 °C–25 °C) and humidity (40%–50%). To establish the xenograft model in nude mice, we selected HeLa cells, which exhibit an easier tendency for tumor formation *in vivo*. HeLa cells (1 × 10^6^) in the log-phase were harvested and administered at a volume of 100 µL per injection site into the right flank of each mouse. Treatment was initiated 2 weeks after tumor implantation and continued for a duration of 28 days. Mice were randomly allocated into three groups (n = 6 per group) and treated with intraperitoneal injections of PEC at 25 mg/kg every 2 days or 50 mg/kg every 2 days, respectively, with the DMSO-treated group serving as the control. Mice within the same treatment group were housed together in a single cage. Tumor dimensions were assessed using a non-digital sliding calipers, and the tumor volume was calculated as 0.5 × length × width^2^. Tumor volume was quantified weekly using a sliding caliper. Mice were humanely euthanized by cervical dislocation on day 29, followed by the collection and weighing of tumor tissues.

#### 2.3.11 Haematoxylin and eosin (H&E) staining

The hearts, livers, and other organs were harvested from mice immediately after the experiments concluded. These tissues were fixed overnight in a 4% paraformaldehyde solution before being embedded in paraffin. Following this, 3-µm tissue sections were prepared, deparaffinized, and subjected to H&E staining. Microscopic images were then captured using a microscope.

#### 2.3.12 Statistical analysis

All experiments were conducted in triplicate, and representative results from these repetitions were presented. Statistical significance between groups was evaluated using Student’s t-test or one-way ANOVA. Data analysis was performed using SPSS version 26.0 and GraphPad Prism version 9.0. The mean values were reported as the ‘center values’, with error bars representing the standard deviation (SD). Statistical significance was indicated as follows: *p < 0.05, **p < 0.01.

## 3 Results

### 3.1 Network pharmacology prediction

#### 3.1.1 Identification of PEC targets against CC and PPI network

In this study, we identified a total of 112 potential targets for PEC using SwissTargetPrediction and the Traditional Chinese Medicine Systems Pharmacology Database (TCMSP). Specifically, 100 targets were obtained from SwissTargetPrediction and an additional 12 from TCMSP. After removing redundant entries, 108 unique PEC-associated targets were retained. For CC-related targets, human-specific (*H. sapiens*) genes were retrieved from the GeneCards database (yielding 9,872 targets) and the OMIM database (199 targets). Following the removal of duplicates, 10006 distinct CC-related targets were confirmed (Supplementary Figure S1).

**FIGURE 1:**
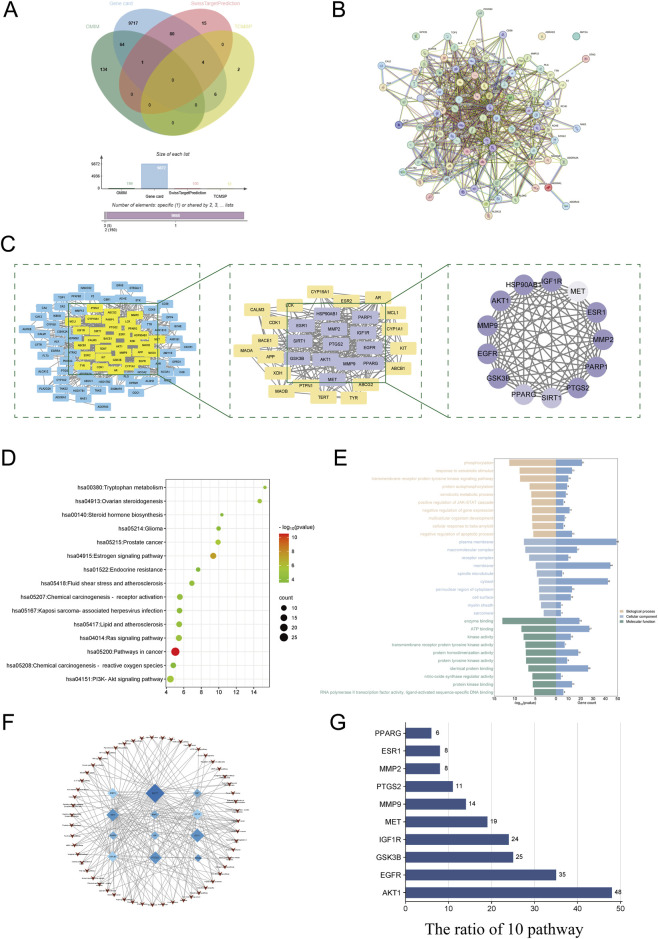
Network pharmacology-based analysis elucidates the impact of PEC on CC. **(A)** the overlapping targets of PEC on CC by Venn diagram. **(B)** PPI network between PEC and CC. **(C)** The filtration process for the primary targets of PEC in relation to key aspects of CC. **(D)** The dot plot visualization of the KEGG pathway analysis illustrates the top 15 pathways along with their corresponding p-values. Dot size reflects gene count per pathway; color intensity indicates significance (darker means lower p-value). **(E)** The top 10 biological characteristics of 83 target genes were identified through GO enrichment analysis. These characteristics include biological process (BP), CeC, and molecular function (MF). **(F)** The establishment of a critical target-pathway network demonstrates the relationships and interactions among different targets and pathways. **(G)** The ratio of principal targets within the leading 10 pathways.

By integrating the target datasets for PEC and CC, a total of 91 common targets were identified (Figure 1A). To elucidate the interrelationships among the shared targets of PEC and CC, a PPI network was constructed and visualized (Figure 1B). With the organism specified as “*H. sapiens*” and interaction score threshold of 0.4 applied to filter significant interactions, this PPI network consisted of 90 distinct nodes, representing the complex interplay among the targets.

These shared targets were imported into Cytoscape software to construct an overlapping network, illustrated in Figure 1A. This network comprised 90 nodes and 679 edges. Nodes with a degree value equal to or greater than twice the median degree of all nodes were selected to form a subnetwork, resulting in a refined structure of 32nodes and 276 edges.

To pinpoint key nodes representing the interaction between PEC and CC, a series of stringent topological analyses were conducted based on degree centrality, betweenness centrality, and closeness centrality. The criteria for selection included degree centrality >16, betweenness centrality >5.951055889, and closeness centrality >0.673913043. These thresholds were set at or above the median values of all nodes in the network. Ultimately, a refined network consisting of 13 critical nodes and 89 connections was established, highlighting the most significant targets involved in the therapeutic action of PEC against CC (Figure 1C).

#### 3.1.2 GO and KEGG pathway enrichment analysis

To elucidate the potential mechanisms of action for PEC in the treatment of CC, we conducted a comprehensive pathway enrichment analysis. A visual representation (Figure 1D) highlights several key signaling pathways implicated in CC, including the “Pathways in Cancer”, AKT signaling pathway, endocrine resistance, Ras signaling pathway, ovarian steroidogenesis, steroid hormone biosynthesis, and tryptophan metabolism. Among these, the “Pathways in Cancer” pathway emerged as particularly relevant, suggesting a significant role in mediating the therapeutic effects of PEC.

GO enrichment analysis was performed using the DAVID 6.7 database to examine the biological processes, molecular functions, and cellular components associated with the common targets of PEC and CC. The GO analysis results (Figure 1E) revealed that the key biological processes involved included phosphorylation, response to xenobiotic stimuli, protein autophosphorylation, and regulation of gene expression. In terms of molecular functions, the common targets were enriched for kinase activity, identical protein binding, ATP binding, enzyme regulation, and transcription factor activity.

The cellular component analysis indicated that these targets were localized in various subcellular regions, including the cytosol, macromolecular complexes, receptor complexes, plasma membrane, and the perinuclear region of the cytoplasm. This comprehensive analysis suggested that PEC’s therapeutic action might be mediated through diverse molecular functions and biological processes, impacting multiple signaling pathways and cellular compartments relevant to CC progression.

To better understand the involvement of these targets in relevant biological pathways, a target-pathway network diagram was generated using Cytoscape 3.9.2 software (Figure 1F). The analysis highlighted the key nodes, which were ranked based on their participation in multiple signaling pathways. Notably, AKT1 emerged as a central node, showing involvement in various critical pathways, underscoring its potential as a pivotal target for the therapeutic action of PEC against CC (Figure 1G).

### 3.2 Molecular docking

Molecular docking simulations were performed using Discovery Studio 3.9.2 to evaluate the interaction between AKT1 and PEC. In docking studies, a binding energy less than zero indicates that the ligand can freely associate with the receptor, with lower binding energy values reflecting stronger binding affinities. As shown in Table 1, the binding energies for different conformations of PEC with the AKT1 target ranged from −6.4 to −8.0 kcal/mol, indicating a strong binding affinity between PEC and AKT1. Its binding energy to AKT is similar to that of the well-characterized AKT inhibitor MK2206 (−6.7 to −8.7 kcal/mol) (Table 2). This robust interaction was attributed to the formation of stabilizing bonds, including hydrogen bonds and hydrophobic interactions.

**TABLE 1:** The energy of different modes of PEC-AKT and their corresponding sites analysis.

Mode	Affinity (kcal/mol)	Dist from best mode (rmsd l.b.| rmsd u.b.)	
1	−8	0	0
2	−7.9	5.99	8.271
3	−7.9	2.134	2.518
4	−7.9	5.035	8.111
5	−7.8	6.408	8.673
6	−7.7	4.352	8.248
7	−7.7	5.902	8.416
8	−7.6	1.874	2.228
9	−7.6	6.362	7.842
10	−7.6	5.241	8.649
11	−7.3	3.724	8.51
12	−7.1	5.2	8.139
13	−7.1	4.073	4.284
14	−7	6.341	7.331
15	−7	19.183	20.752
16	−7	4.059	4.495
17	−6.7	5.42	7.036
18	−6.7	19.613	20.319
19	−6.5	19.701	20.621
20	−6.4	19.034	20.812

^a^ Root Mean Square Deviation (RMSD) is a quantitative measure for assessing spatial similarity of molecular structures, extensively applied in structural biology, computational chemistry, and molecular dynamics.

^b^ In structural analysis, rmsd l.b (lower bound) and rmsd u. b (upper bound) define the permissible range of Root Mean Square Deviation between molecular structures.

^c^ The lower bound sets the minimum acceptable deviation, while the upper bound specifies the maximum allowable deviation.

**TABLE 2:** The energy of different modes of MK2206-AKT and their corresponding sites analysis.

Mode	Affinity (kcal/mol)	Dist from best mode (rmsd l.b.| rmsd u.b.)	
1	−8.7	0	0
2	−8.6	3.089	5.849
3	−8.6	4.122	8.404
4	−8.5	2.941	7.887
5	−8.4	5.681	7.915
6	−8.4	3.432	5.917
7	−8.4	4.122	7.806
8	−8.2	4.720	8.139
9	−8.2	4.055	7.677
10	−8.0	4.575	7.164
11	−8.0	4.955	8.959
12	−7.9	4.362	8.116
13	−7.8	4.006	7.246
14	−7.7	4.209	8.799
15	−7.5	4.119	6.079
16	−7.4	4.589	6.803
17	−6.9	21.080	24.194
18	−6.8	22.090	23.843
19	−6.8	4.590	8.780
20	−6.7	4.002	7.133

**FIGURE 2:**
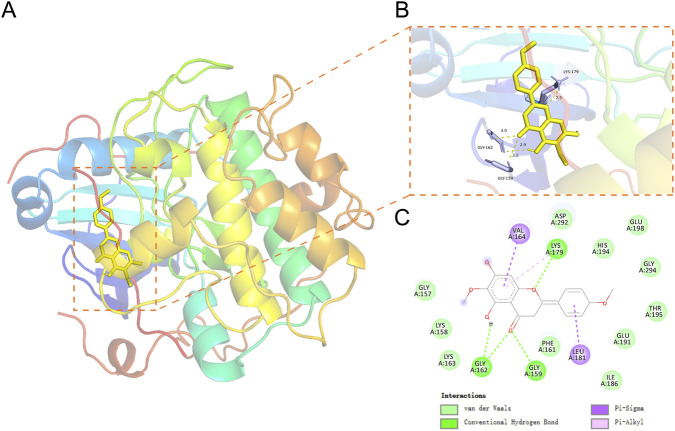
Molecular representations of PEC interacting with its anticipated protein targets. **(A)** Docking of the crystal structure of AKT1 (PDB ID:3qkl) in three-dimensional (3D) modeling. **(B)** 3D of the docking process within the crystal structure of PEC. **(C)** PEC docking 2D model.

The molecular docking analysis (Figures 2A–C) revealed that PEC bound to AKT1 via multiple interactions. Key residues involved in van der Waals forces included Asp292, His194, Glu198, Gly294, Thr195, Glu191, Ile186, Phe161, Lys163, Lys158, and Gly157. Additionally, strong hydrogen bonds were formed at the Gly162, Gly159, and Lys179 residues, whereas MK2206 formed hydrogen bonds with the Glu198, Lys179, THR5(Supplementary Figure S2)These interactions collectively contributed to the enhanced stability and strong affinity of PEC for AKT1, suggesting a potential mechanism for its therapeutic efficacy against CC.

### 3.3 Cell experiments

#### 3.3.1 PEC inhibits proliferation and metastasis of CC cells

**FIGURE 3:**
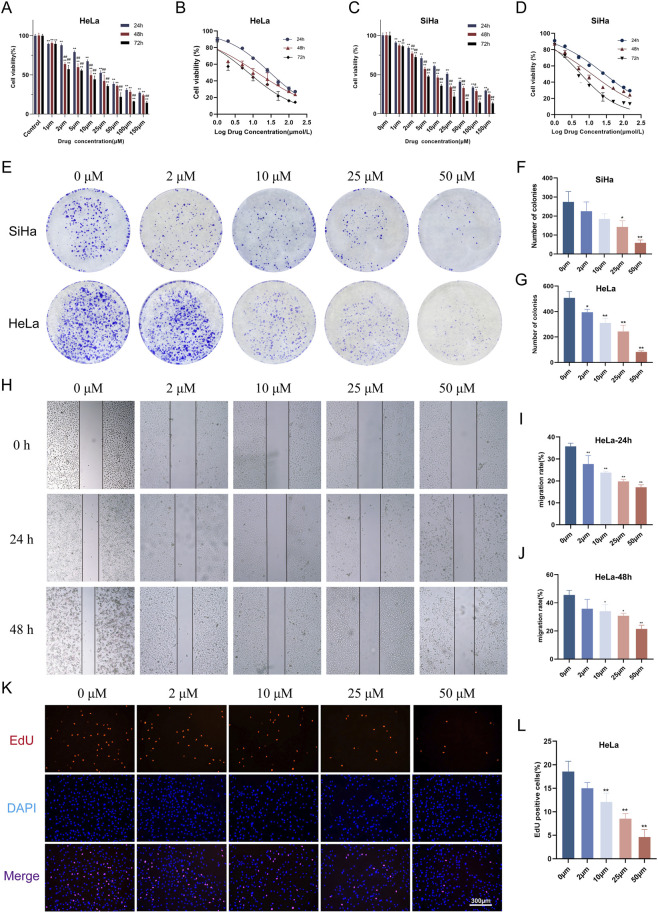
PEC demonstrates anti-tumor properties in human CC cell lines and anti-migration effects in HeLa cell lines. **(A–D)** The CCK8 assay of HeLa and SiHa cells treated with various concentrations (0, 1, 2, 5, 10, 25, 50, 100, and150 μM) of PEC for 24, 48, and 72 h **(E–G)** An investigation into the capacity for colony formation in HeLa and Siha cells subjected to treatment with PEC (0, 5,10, 25, and 50 μM). The evaluation of cell density was performed utilizing ImageJ software. **(H–J)** The wound healing assessment was conducted on HeLa cells that were treated with PEC (0, 5,10, 25, and 50 μM). The migration rates for both 24-h and 48-h intervals were evaluated using ImageJ software. **(K)** EdU staining was employed to evaluate the proliferative capacity of HeLa cells at different concentrations of PEC. **(L)** The analysis of fluorescence intensity was conducted utilizing ImageJ software. Mean ± SD, n ≥ 3; *P < 0.05; **P < 0.01 vs. the control group. #P < 0.05; ##P < 0.01 vs. the 24-h group.

To evaluate the effects of PEC on the proliferation of CC cells, we conducted preliminary experiments using varying concentrations of PEC, followed by assessment of its impact on the viability of HeLa and SiHa cells using the CCK-8 assay. As shown in Figures 3A,C, PEC demonstrated a significant inhibitory effect on cervical cancer cells, which was both time- and dose-dependent. Further analysis determined the 24-h IC_50_ values for PEC in HeLa and SiHa cells, calculated as 29.77 µM and 27.74 µM, respectively. The 48-h IC_50_ values of PEC were determined as 12.96 µM in HeLa cells and 11.84 µM in SiHa cells, while the 72-h IC_50_ values decreased to 7.32 µM and 5.775 µM, respectively (Figures 3B,D).

**FIGURE 4:**
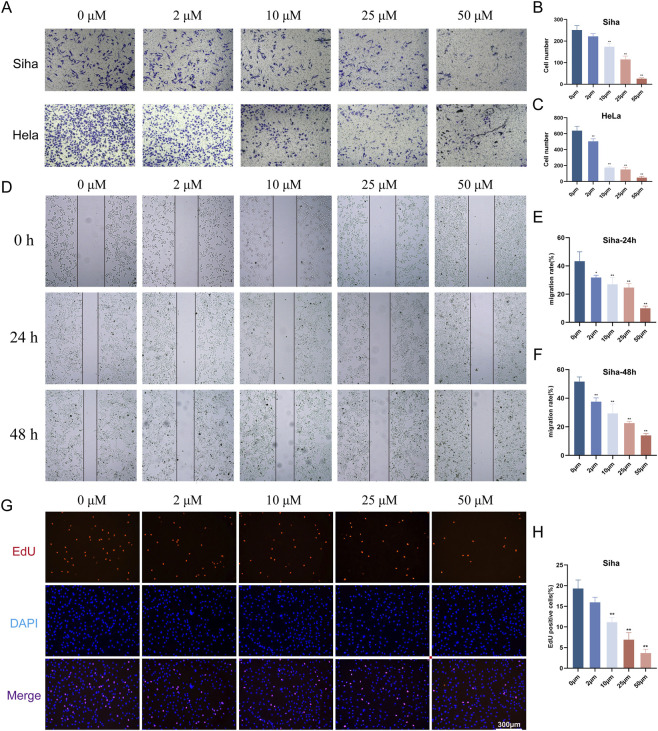
PEC demonstrates anti-invasive properties in human CC cell lines and inhibits migration in SiHa cells. **(A–C)** HeLa and SiHa cells were treated with PEC at the indicated concentration (0, 2, 10, 25, and 50 μM) for 24 h and evaluated by the invasion assay. The evaluation of cell numbers was performed utilizing ImageJ software. **(D–F)** The wound healing assessment was conducted on SiHa cells that were treated with PEC (0, 5,10, 25, and 50 μM). The migration rates for both 24-h and 48-h intervals were evaluated using ImageJ software. **(G)** EdU staining was employed to evaluate the proliferative capacity of SiHa cells at different concentrations of PEC. **(H)** The analysis of fluorescence intensity was conducted utilizing ImageJ software. Mean ± SD, n ≥ 3; *P < 0.05; **P < 0.01 vs. the control group.

The clone formation assay revealed a marked decrease in the number of cell colonies with increasing concentrations of PEC (Figures 3E–G), suggesting a strong inhibitory effect on cell proliferation. Additionally, the EdU assay was employed to further assess the impact of PEC on cell proliferation at different concentrations. Results from this assay (Figures 3K,L, 4G,H) indicated a significant reduction in EdU-positive cells in both HeLa and SiHa lines, consistent with the findings from the CCK-8 assay, confirming that PEC effectively inhibited cell proliferation in a concentration-dependent manner.

Moreover, transwell invasion assays showed a notable decrease in the invasive capacity of HeLa and SiHa cells following treatment with PEC (Figures 4A–C). The wound healing assay further demonstrated that PEC suppressed the migratory ability of CC cells at all tested concentrations, compared to the untreated control group (Figures 3H–J, 4D–F). These results collectively suggested that PEC exhibited strong anti-tumor activity by impairing both the proliferative and metastatic potential of CC cells. Given these findings, we proceeded to investigate the underlying molecular mechanisms through which PEC exerted its growth-inhibitory effects in CC cells, aiming to uncover the pathways involved in its anticancer activity.

#### 3.3.2 PEC induces apoptosis in HeLa and SiHa cells

Apoptosis, a programmed cell death mechanism essential for cellular homeostasis, is frequently dysregulated in malignancies to sustain tumor survival (Carneiro and El-Deiry, 2020; Zhang et al., 2021; Newton et al., 2024). Pharmacological restoration of apoptotic signaling represents a strategic anticancer approach (Hanahan and Weinberg, 2011). To determine whether PEC could trigger apoptosis in HeLa and SiHa cells, we conducted a series of assays to evaluate its pro-apoptotic effects.

**FIGURE 5:**
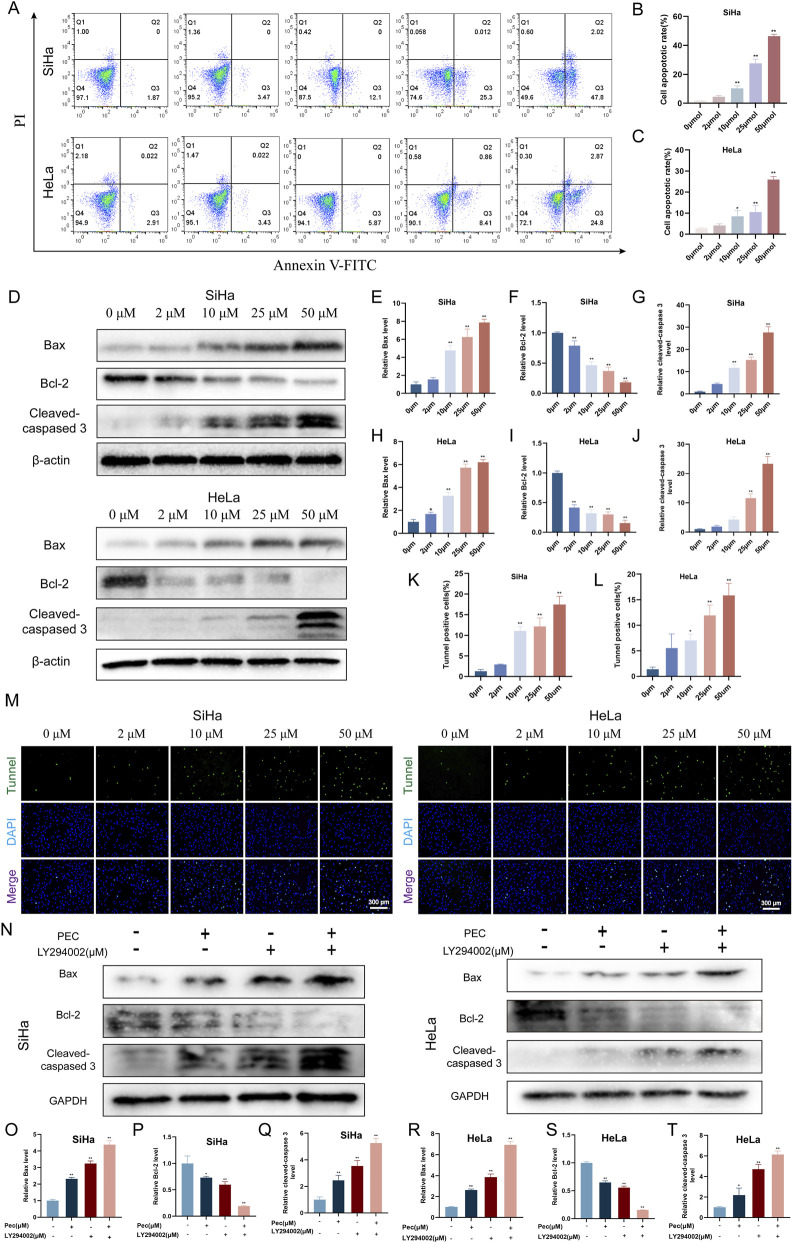
PEC induces programmed cell death in human CC cells. **(A)** The apoptosis assay was performed on HeLa and SiHa cells treated with PEC (0, 5, 10, 25, and 50 μM). The stained cells were analyzed using flow cytometry after being labeled with the Annexin V-FITC/PI detection kit. **(B,C)** Percentage of apoptosis was quantified using FlowJo software. **(D)** Western blotting analysis was conducted to evaluate the expression levels of Bax, Bcl-2, and cleaved caspase-3 proteins in HeLa and SiHa cells subjected to treatment with PEC (0, 5, 10, 25, and 50 μM); **(E–J)** The quantification of protein expression levels for Bcl-2, Bax, and cleaved caspase-3 was conducted utilizing ImageJ software. **(K,L)** ImageJ software was utilized to quantify the percentage of cells exhibiting positive TUNEL staining. **(M)** The TUNEL assay was employed to assess the apoptosis of HeLa and SiHa cells. **(N)** Western blotting analysis was conducted to evaluate the expression levels of Bax, Bcl-2, and cleaved caspase-3 proteins in HeLa and SiHa cells subjected to treatment with PEC and LY294002. **(O–T)** Protein expression levels of Bcl-2, Bax, and cleaved caspase-3 were quantified using ImageJ software. Mean ± SD, n ≥ 3; *P < 0.05; **P < 0.01 vs. the control group.

In PEC-treated cells, we observe chromatin condensation, plasma membrane blebbing, and cell shrinkage (Figures Supplementary Figure S4). Western blotting analysis revealed that treatment with PEC for 24 h resulted in upregulation of Bax (a pro-apoptotic protein) and cleaved caspase-3 (a key effector enzyme in apoptosis), along with a concomitant downregulation of Bcl-2 (an anti-apoptotic protein) (Figures 5D–J). Furthermore, our analysis revealed that CC cells displayed reduced mitochondrial membrane potential, demonstrated by the characteristic red-to-green fluorescence transition observed post-PEC treatment using JC-1 staining (Supplementary Figure S3). These findings suggest that PEC induces apoptosis through both regulation of apoptotic markers and mitochondrial depolarization.

Further assessment using the TUNEL assay demonstrated a significant increase in the percentage of TUNEL-positive cells following PEC treatment. In SiHa cells, the proportions of apoptotic cells were 1.25%, 2.94%, 11.06%, 12.17%, and 17.49% at PEC concentrations of 0 μM, 2 μM, 10 μM, 25 μM, and 50 μM, respectively (Figures 5K–M). Compared to the control group (0 µM), the percentage of apoptotic cells increased by 1.69%, 9.82%, 10.90%, and 16.25% at escalating PEC doses. In HeLa cells, the TUNEL-positive rates were 1.41%, 5.53%, 7.03%, 11.94%, and 15.83% for the same PEC concentrations, corresponding to increases of 4.12%, 5.52%, 10.53%, and 14.42% relative to the control group.

To further confirm the induction of apoptosis by PEC, flow cytometry analysis using Annexin V-FITC/PI double staining was performed on HeLa and SiHa cells treated with PEC for 24 h (Figures 5A–C). In SiHa cells, the apoptotic rates increased significantly to 3.47%, 12.1%, 25.3%, and 47.8% at PEC concentrations of 2 μM, 10 μM, 25 μM, and 50 μM, respectively. Similarly, HeLa cells exhibited apoptotic rates of 3.43%, 5.87%, 8.41%, and 24.8% at the same concentrations. In contrast, the negative control group showed baseline apoptosis rates of 2.91% for HeLa cells and 1.87% for SiHa cells.

#### 3.3.3 PEC induces autophagy in HeLa and SiHa cells

Autophagy, a cellular process crucial for maintaining homeostasis, plays a significant role in tumor development and is increasingly recognized as a promising target for cancer therapy (Li et al., 2020). To investigate the effects of PEC on autophagy in CC cells, we conducted a series of experiments using HeLa and SiHa cell lines.

**FIGURE 6:**
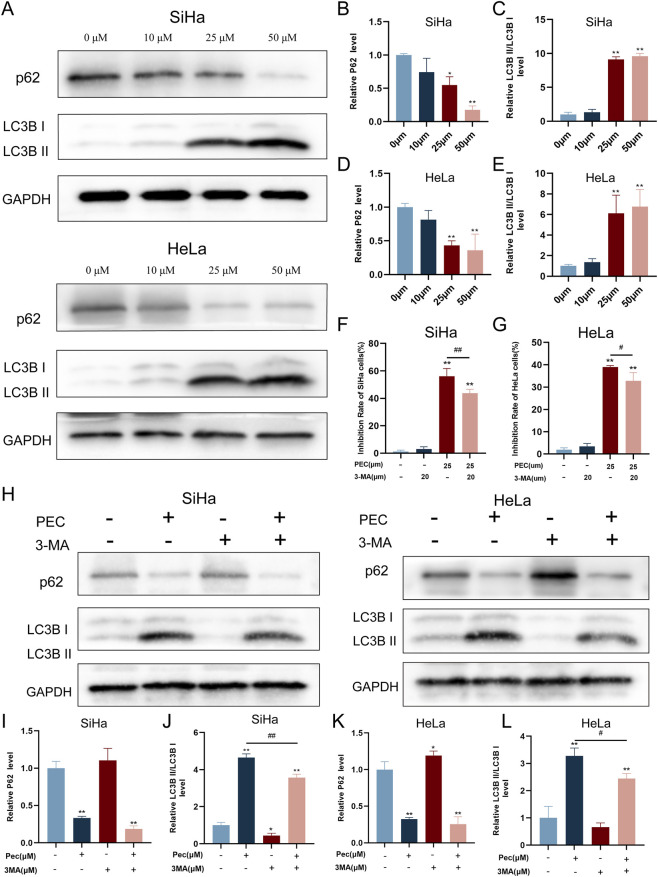
PEC induces autophagy in CC cells. **(A)** Western blotting analysis was conducted to evaluate the expression levels of p62 and LC3B II proteins in HeLa and SiHa cells subjected to treatment with PEC (0, 10, 25, and 50 μM); **(B–E)** The protein expression levels of p62 and LC3B II were quantified using ImageJ software. **(F,G)** The CCK8 assay of HeLa and SiHa cells treated with PEC (25 μM) and 3-MA (20 μM). **(H)** Western blotting analysis was conducted to evaluate the expression levels of p62 and LC3B II proteins in HeLa and SiHa cells subjected to treatment with PEC and 3-MA. **(I–L)** Protein expression levels of p62 and LC3B II were calculated using ImageJ software. Mean ± SD, n ≥ 3; *P < 0.05; **P < 0.01 vs. the control group. #P < 0.05; ##P < 0.01 vs. the group treated with PEC only.

Western blotting analysis showed that treatment with PEC led to a substantial increase in the expression ratio of LC3B II/LC3B I, a well-established marker of autophagy, while simultaneously decreasing the expression of p62, a protein that is degraded during autophagic processes (Figures 6A–E). These changes indicated the activation of autophagy following PEC treatment.

Furthermore, the addition of 3-MA, an established autophagy inhibitor, effectively reduced the extensive cell death induced by PEC (25 µM), suggesting that autophagy contributed to the cytotoxic effects of PEC (Figures 6F,G). Treatment with 3-MA alone (20 µM) resulted in a decreased expression of LC3B II/LC3B I, confirming its role in inhibiting autophagic flux. In subsequent experiments, HeLa and SiHa cells were co-treated with 3-MA (50 µM) and PEC (25 µM). The combined treatment led to a reduction in the expression levels of LC3B II/LC3B I compared to PEC treatment alone, consistent with previous findings by Liu et al. (Liu et al., 2022) (Figures 6H–L). This observation further supported the notion that PEC induced autophagy in CC cells and that inhibiting autophagy could mitigate PEC-induced cytotoxicity. Overall, these results suggested that the anti-cancer effects of PEC on HeLa and SiHa cells might be closely linked to its ability to induce autophagy, highlighting its potential as a natural therapeutic agent targeting autophagic pathways in CC treatment.

#### 3.3.4 PEC modulates the AKT/mTOR signaling pathway to induce apoptosis and autophagy in HeLa and SiHa cells

Network pharmacology predictions suggested that AKT was a direct molecular target of PEC. The AKT/mTOR signaling axis is pivotal in regulating cell proliferation and survival, making it a key pathway in oncogenesis (O'Donnell et al., 2018). Consequently, targeting the AKT/mTOR pathway is considered a promising strategy for developing novel cancer therapies (Yu et al., 2022).

**FIGURE 7:**
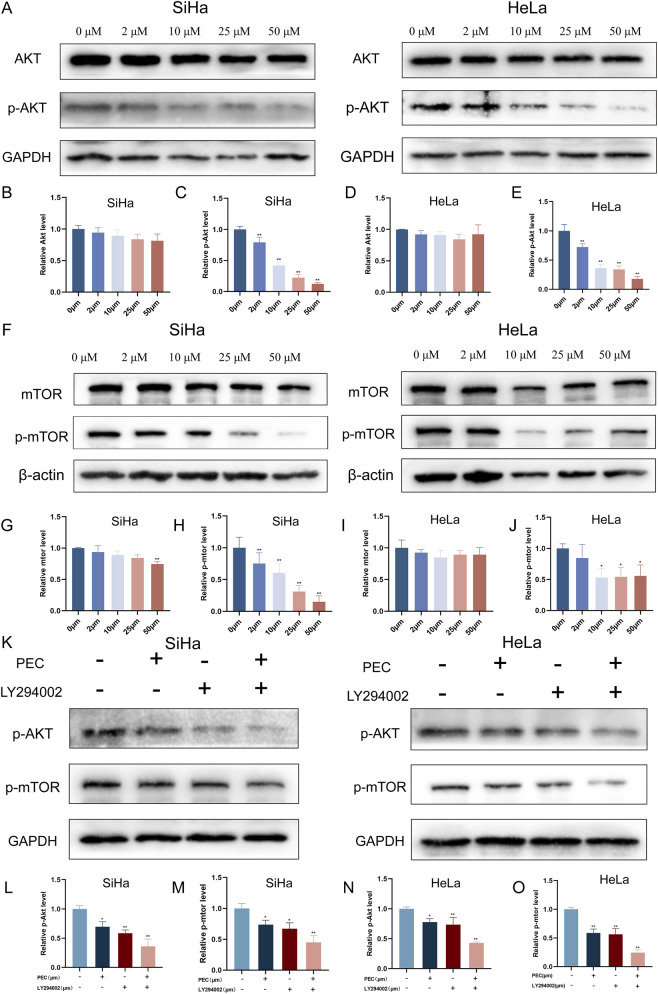
The PEC modulates the AKT/mTOR signaling pathway to induce apoptosis and autophagy in CC cells. **(A)** Western blotting analysis was conducted to evaluate the expression levels of AKT and p-AKT proteins in HeLa and SiHa cells subjected to treatment with PEC (0, 5, 10, 25, and 50 μM); **(B-E)** The quantification of protein expression levels for AKT and p- AKT was conducted utilizing Image J software. **(F)** Western blotting analysis was conducted to evaluate the expression levels of mTOR and p-mTOR proteins in HeLa and SiHa cells subjected to treatment with PEC (0, 5, 10, 25, and 50 μM). **(G-J)** Protein expression levels of mTOR and p-mTOR were quantified using Image J software. **(K)** Western blotting analysis was conducted to evaluate the expression levels of p-AKT and p-mTOR proteins in HeLa and SiHa cells subjected to treatment with PEC and LY294002. **(L-O)** The quantification of protein expression levels for p-AKT and p- mTOR was conducted utilizing Image J software. Mean ± SD, n ≥ 3; *P < 0.05; **P 0.01 vs. the control group.

Our experimental data demonstrated that PEC treatment resulted in a significant, dose-dependent reduction in the phosphorylation of AKT at the Ser473 residue after 24 h (Figures 7A–E). This indicated that PEC effectively inhibited AKT activation. In parallel, we investigated the impact of PEC on mTOR, a downstream effector of AKT. Western blotting analysis revealed that PEC exposure led to a marked decrease in the phosphorylated form of mTOR (Ser2448) in both HeLa and SiHa cell lines (Figures 7F–J).

To further elucidate the role of the AKT/mTOR pathway in PEC-mediated effects, we utilized the PI3K inhibitor LY294002, which specifically inhibits the AKT/mTOR signaling cascade. The results showed that both PEC and LY294002 independently suppressed the phosphorylation of AKT and mTOR, indicating similar inhibitory effects on this oncogenic pathway (Figures 7K–O). Moreover, the combined treatment with PEC (25 µM) and LY294002 (80 µM) exhibited a synergistic effect, leading to a more pronounced reduction in the expression levels of phosphorylated AKT (p-AKT) and phosphorylated mTOR (p-mTOR) compared to either treatment alone (Figures 7K–O).

These findings suggested that PEC exerted its anti-cancer effects in HeLa and SiHa cells by inducing both autophagy and apoptosis, primarily through the suppression of the AKT/mTOR signaling pathway. This dual induction of cell death highlighted PEC’s potential as a promising natural therapeutic agent for targeting CC.

Moreover, previous studies have reported that the PI3K inhibitor LY294002 significantly enhances apoptosis when combined with other pro-apoptotic agents in HeLa and SiHa cells (Liu et al., 2022). Consistent with these findings, our Western blotting analysis indicated that co-treatment with PEC (25 µM) and LY294002 (80 µM) led to a further reduction in Bcl-2 expression, along with increased levels of Bax and cleaved caspase-3 compared to treatment with PEC or LY294002 alone (Figures 5N–T). Collectively, these results demonstrated that PEC effectively induced apoptosis in HeLa and SiHa cells in a dose-dependent manner, underscoring its potential as a natural anticancer agent.

### 3.4 PEC inhibits the growth of hela cells in the tumor model

**FIGURE 8:**
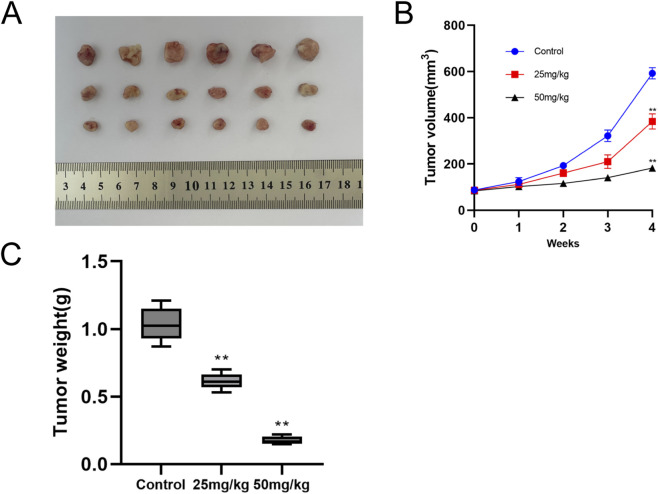
Pectolinarigenin inhibits the growth of cervical cancer in the xenograft mouse model. **(A)** Representative images of cervical cancer grafts excised from mice following 28 days of pectolinarigenin administration. **(B)** Summary of tumor volume in the control and pectolinarigenin-treated groups in the xenograft vivo’s model (**P < 0.01). **(C)** Tumor weight in each group was quantified (**P < 0.01).

**FIGURE 9:**
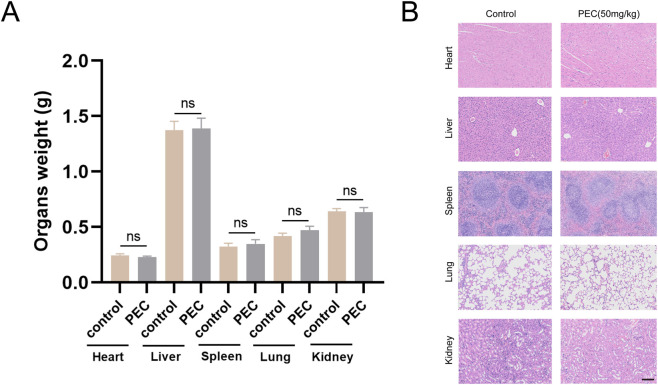
The potential toxic effects of pectolinarigenin in mice. **(A)** The weights of major organs were assessed at the conclusion of the experiment. ns:no significance. **(B)** Major organs from control group and pectolinarigenin-treated group were stained with H&E. Scale bar, 100 µm.

By comparing the differences in tumor growth rates between the control group and the PEC-treated groups, the results demonstrated that the PEC treatment significantly inhibited tumor volume growth, with the inhibitory effect exhibiting a dose-dependent pattern, increasing as the drug dosage increased (Figure 8). To investigate the potential toxic effects of PEC, female BALB/c mice were administered intraperitoneal injections of the compound at a dose of 50 mg/kg every 2 days over a 28-day period. Body weight was monitored weekly throughout the study. On day 29, the mice were euthanized, and their major organs were collected and weighed. The data showed no significant differences in organ weight between the treated and control groups (Figure 9A). Histopathological analysis using H&E staining also revealed no notable structural damage in the heart, lung, liver, spleen, or kidney (Figure 9B). These findings demonstrate that PEC not only inhibits tumor growth but also does not induce significant adverse effects in mice.

## 4 Discussion

CC remains a leading gynecological malignancy worldwide, with advanced stages often requiring radiotherapy and chemotherapy due to high recurrence and metastasis rates (Cao et al., 2024; Serkies and Jassem, 2018; Gupta et al., 2018). However, treatment efficacy is limited by severe side effects and drug resistance (Gupta et al., 2018). Natural compounds are increasingly investigated as safer alternatives, demonstrating anti-cancer effects through cell cycle modulation, apoptosis induction, and autophagy regulation (Qian et al., 2024; Lee et al., 2018; Yao et al., 2024). PEC, a flavonoid derived from citrus plants and Radix Cirsii Japonic, exhibits multi-target therapeutic potential including anti-inflammatory, antioxidant, and immunomodulatory properties. While its safety has been confirmed in various cell models, the specific mechanisms underlying PEC’s anti-CC effects remain insufficiently investigate, underscoring the need for further research in this area (Qian et al., 2024; Lee et al., 2018; Yao et al., 2024).

Network pharmacology integrates data mining and network modeling to identify drug targets and mechanisms. Through topological analysis, we identified 13 pivotal targets of PEC in CC treatment. To further explore the potential mechanisms of action, we conducted KEGG and GO enrichment analyses. The results highlighted several keys signaling pathways associated with PEC, including the PI3K-AKT pathway, Ras signaling, the “pathway in cancer”, and the estrogen signaling pathway. Notably, the “pathway in cancer” emerged as the most influential, encompassing multiple critical signaling cascades, such as JAK-STAT, HIF-α, and AKT-mTOR pathways, all of which play essential roles in regulating cancer cell pathophysiology.

Our GO enrichment analysis revealed that PEC significantly impacted protein phosphorylation via enzyme binding, suggesting its potential role in modulating the progression of CC. A deeper examination of the proteins involved in these pathways identified AKT1 as a central hub in PEC’s anti-cancer mechanism. AKT, a serine-threonine kinase, regulates cellular proliferation, survival, and apoptosis under physiological conditions (Revathidevi and Munirajan, 2019; Hua et al., 2021). However, its aberrant activation in cancers drives apoptosis resistance, invasiveness, and metastasis (Hua et al., 2021; McKenna et al., 2020; Fulda, 2014). Mechanistically, AKT phosphorylates TSC2 to destabilize T1462, relieving mTOR inhibition and promoting tumor growth via eukaryotic translation initiation (Hua et al., 2021; Ha et al., 2020; Fu and Wu, 2023). Molecular docking simulations, which utilize geometric and energetic matching to predict drug-target interactions, revealed that PEC bound favorably to AKT1, with binding energies ranging from −6.4 to −8.0 kcal/mol (Table 1). This strong binding affinity suggested that PEC might exert its therapeutic effects in CC through modulation of the AKT signaling pathway. Overall, these findings indicated that PEC’s anticancer effects are primarily mediated by suppression of the AKT/mTOR signaling axis, providing a mechanistic foundation for its potential use as a natural CC therapeutic. To validate these predictions, *in vitro* analyses confirmed that PEC markedly suppressed AKT phosphorylation at Ser473 and downstream mTOR activation in CC cells, effectively inhibiting pathway hyperactivation and highlighting its therapeutic potential as an AKT/mTOR axis inhibitor. Functional assays revealed that PEC not only suppressed the proliferation of HeLa and SiHa cells but also diminished their metastatic and invasive capabilities. Previous studies have established that a reduction in AKT activity is a key trigger for the initiation of programmed cell death, particularly apoptosis (Revathidevi and Munirajan, 2019; McKenna et al., 2020). Apoptosis can occur via two main pathways: the intrinsic (mitochondrial) pathway and the extrinsic (death receptor) pathway. In this study, we elucidated the mechanism by which PEC induces apoptosis in CC cells, specifically targeting the intrinsic mitochondrial pathway regulated by the AKT/mTOR axis. While the intrinsic pathway is classically characterized by the dynamic regulation of Bcl-2 family proteins and subsequent mitochondrial outer membrane permeabilization (Carneiro and El-Deiry, 2020; Obeng, 2021), our results explicitly demonstrate that PEC treatment disrupts this balance. We observed a significant upregulation of the pro-apoptotic effector Bax, which facilitates the release of apoptogenic factors and the subsequent activation of the executioner caspase-3. The elevated levels of cleaved caspase-3 in PEC-treated cells confirm the irreversible commitment to apoptosis and the dismantling of cellular structures (Newton et al., 2024; Asadi et al., 2022). Furthermore, we validated the upstream regulation of this cascade by investigating the synergistic effects of PEC and LY294002, a PI3K inhibitor. Since the AKT/mTOR signaling pathway is a critical survival axis that inhibits apoptosis, its suppression is a key therapeutic target. Our data showed that co-administration of PEC and LY294002 not only profoundly inhibited p-AKT and p-mTOR levels but also synergistically enhanced the expression of Bax and cleaved caspase-3 compared to individual treatments. These findings confirm that PEC activates the mitochondrial apoptotic cascade in CC cells primarily by acting as a potent inhibitor of the AKT/mTOR signaling pathway, thereby overcoming the survival signals that normally suppress BAX/BAK oligomerization.

Beyond its role in driving apoptosis, the AKT/mTOR axis also serves as a master regulator of autophagy (Asadi et al., 2022; Cao et al., 2021). Consequently, we hypothesized that the PEC-mediated inhibition of this pathway would concurrently trigger autophagic activity. Consistent with this hypothesis, PEC treatment induced a dose-dependent increase in the LC3B-II/LC3B-I ratio and a concurrent reduction in p62 levels. These biochemical changes indicate that PEC successfully initiates autophagic flux, likely by relieving the inhibitory phosphorylation exerted by mTORC1 (Cao et al., 2021; Vieira et al., 2014). Given that autophagy can function paradoxically as either a cytoprotective survival mechanism or a cytotoxic death pathway depending on the context (Gewirtz, 2014; Bai et al., 2022), it was crucial to define its specific role in our study. To achieve this, we employed the autophagy inhibitor 3-MA. Our results demonstrated that co-treatment with 3-MA significantly attenuated the cell death induced by PEC in HeLa and SiHa cells. While 3-MA effectively blocked the autophagic process (evidenced by the reduced LC3B-II/I ratio compared to PEC alone), the key finding is that this inhibition restored cell viability. This aligns with observations in Rosa rugosa polysaccharides (Liu et al., 2022) and confirms that PEC-induced autophagy contributes to cell death rather than survival. Collectively, these data suggest that PEC acts as a dual-targeting agent, enhancing cytotoxicity by simultaneously triggering apoptosis and lethal autophagy through the blockade of the AKT/mTOR signaling pathway.

In summary, our study provided a comprehensive understanding of the molecular targets and mechanisms underlying the anti-cancer effects of PEC on CC, utilizing an integrated approach of network pharmacology and molecular docking analysis. These predictive findings were validated through a series of experiments, establishing a robust theoretical framework for further elucidating the mechanisms through which PEC exerted its anti-CC activity.

While this study established the anti-CC effect and mechanism of PEC, further investigation into its *in vivo* pharmacokinetics and toxicity profile is required to fully assess its therapeutic potential. Although PEC’s *in vitro* IC_50_ might raise questions about clinical translatability, it is important to note that inherent differences exist between *in vitro* and *in vivo* systems. For example, luteolin exhibits significantly higher *in vitro* IC_50_ values in ovarian cancer models than PEC does, yet achieves significant *in vivo* tumor reduction (Li et al., 2023b). Furthermore, advanced drug delivery systems could enhance targeted accumulation at disease sites (>100-fold higher local concentrations vs. free drug) while reducing systemic toxicity (Jeong et al., 2021; Marques et al., 2023; Widder et al., 1978). Additionally, future studies will elucidate the interplay between PEC-induced autophagy and apoptosis in CC cells, and explore its immunomodulatory potential. Given the high prevalence of TP53, AKT, and PTEN mutations in HPV-negative cervical carcinomas (Van Elsland and Neefjes, 2018; Vieira et al., 2014), PEC may emerge as a rational therapeutic strategy for this malignancy. Collectively, these efforts will advance the translational development of this phytochemical for precision CC therapy.

## 5 Conclusion

**FIGURE 10:**
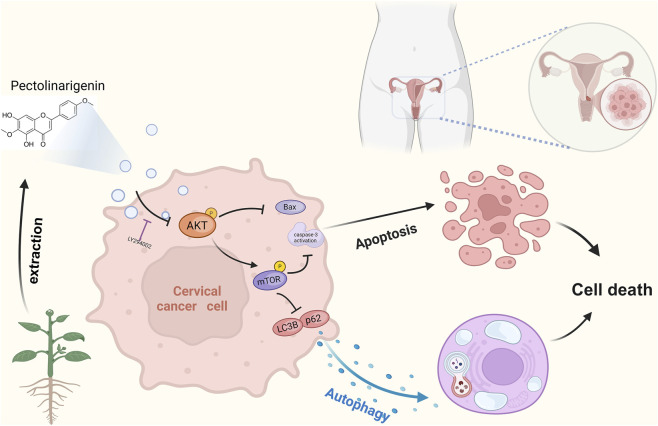
The process of exploring and validating the targets of PEC. Figure created by biorender.

In conclusion, this study provided compelling evidence for the anti-cancer potential of PEC in the treatment of CC, supported by a comprehensive approach combining network pharmacology, molecular docking, and experimental validation. Our analysis identified 13 key molecular targets implicated in PEC’s therapeutic effects, with AKT emerging as the most promising candidate target. Subsequent cell-based experiments confirmed that PEC significantly inhibited the proliferation and migration of both HeLa (cervical adenocarcinoma) and SiHa (cervical squamous carcinoma) cells. Additionally, PEC was shown to robustly induce apoptosis and autophagy, processes that were mechanistically linked to the suppression of AKT/mTOR phosphorylation and the consequent inactivation of the AKT signaling pathway (Figure 10). This study not only underscored the therapeutic potential of PEC as a natural anti-cancer agent but also laid a strong foundation for the future development of PEC-based therapies targeting CC. By advancing our understanding of PEC’s molecular mechanisms, this research opened up promising avenues for the formulation of innovative and effective treatment strategies for CC, offering new hope in the fight against this challenging disease.

## Data Availability

The original contributions presented in the study are included in the article/Supplementary Material, further inquiries can be directed to the corresponding author.
