# Supplementary figures and images for: Targeting the AKT/mTOR axis: pectolinarigenin induces autophagy and apoptosis in human cervical cancer cells
Source: Front Pharmacol. 2026 Mar 17;17:1544170. doi: 10.3389/fphar.2026.1544170 (PMC13036129; doi:10.3389/fphar.2026.1544170)

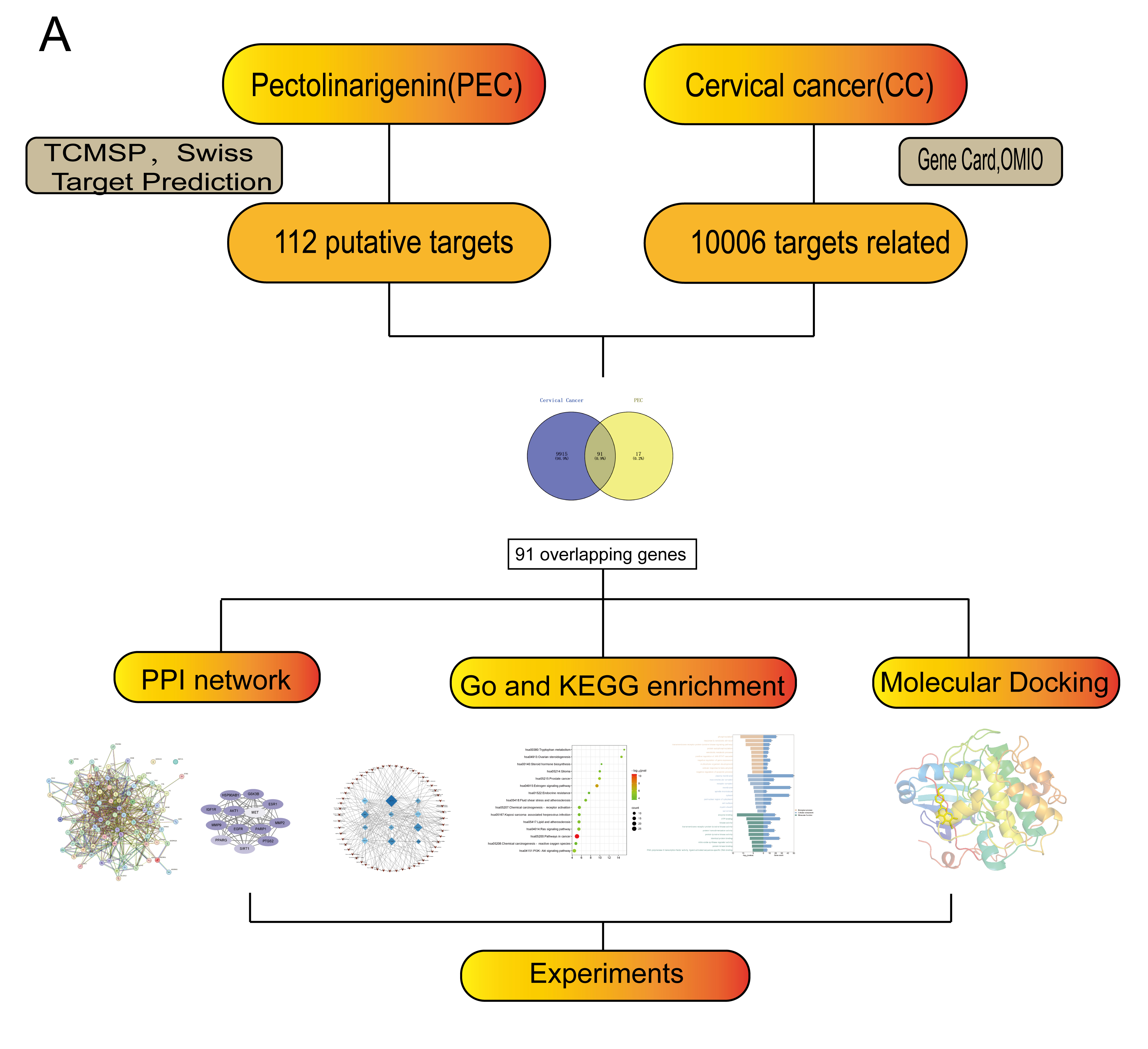

Supplement: Supplementary file 2 [file Image1.jpeg]

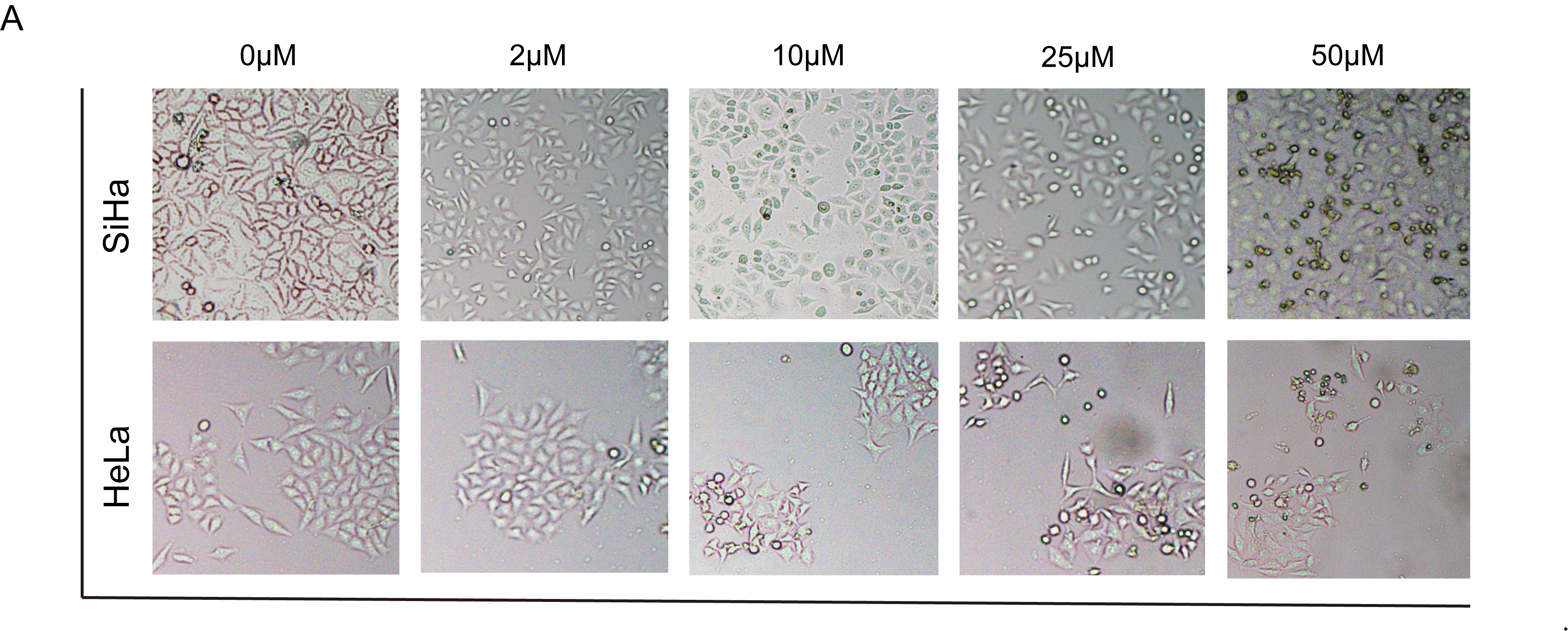

Supplement: Supplementary file 3 [file Image4.jpeg]

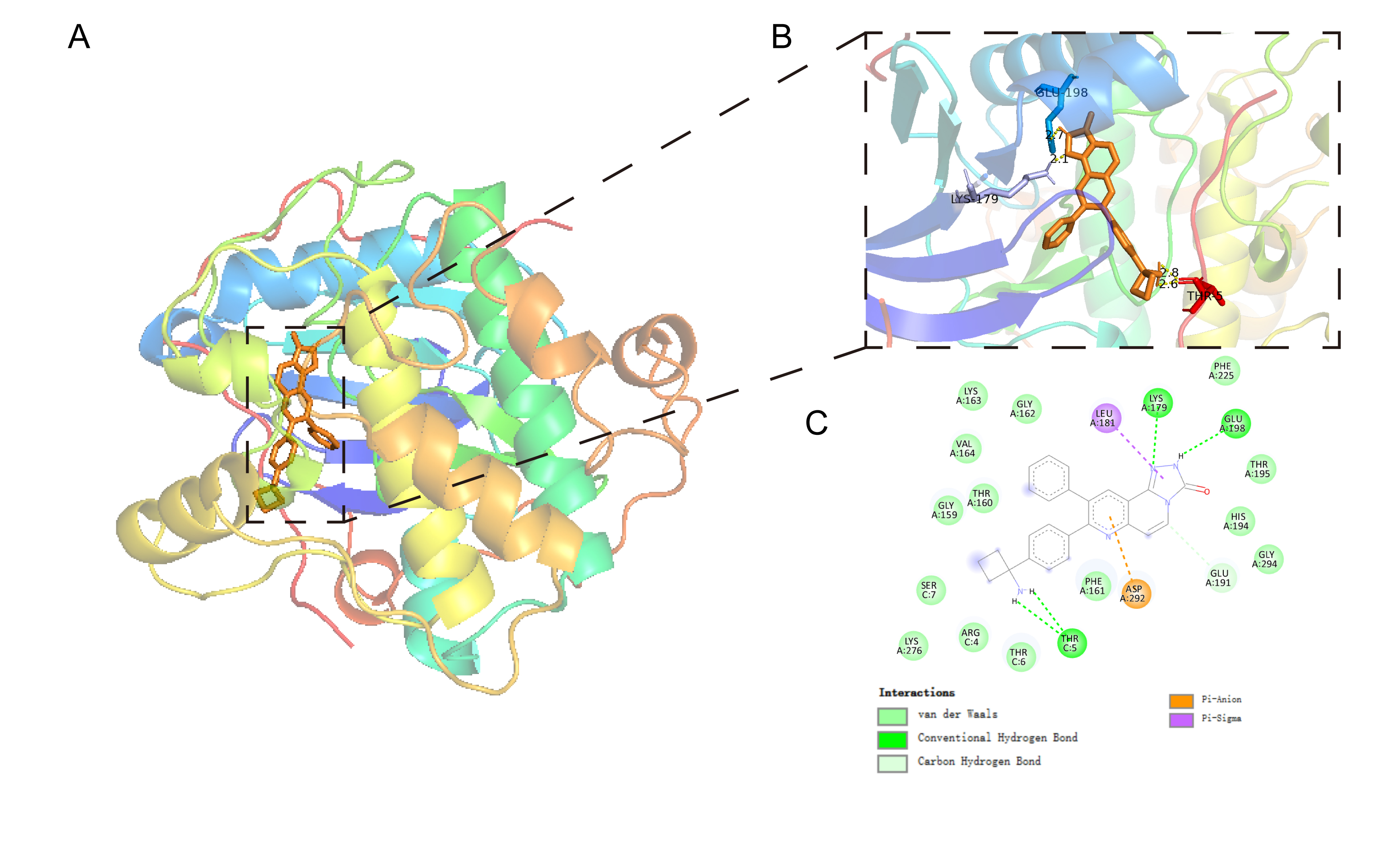

Supplement: Supplementary file 4 [file Image2.jpeg]
